# Clinical outcome of 115 patients with synchronous bilateral Wilms’ tumor: largest cohort of single-center experience

**DOI:** 10.3389/fonc.2025.1630923

**Published:** 2025-10-29

**Authors:** Hosam Y. Asfour, Wael Zekri, M. Hany Hussein, Naglaa Elkinaai, Sarah Sobhy, Mahmoud M. Helmy, Azza Nasr, Amal Refaat, Rana Gamal, Alaa Younes, Moatasem El-Ayadi, Sahar A. Khalil

**Affiliations:** ^1^ Department of Pediatric Oncology, National Cancer Institute, Cairo University, Cairo, Egypt; ^2^ Department of Pediatric Oncology, Children Cancer Hospital Egypt (CCHE), Cairo, Egypt; ^3^ Department of Hematology and Pediatric Oncology, Mouwasat Hospital, Dammam, Saudi Arabia; ^4^ Department of Pathology, National Cancer Institute, Cairo University, Cairo, Egypt; ^5^ Department of Pathology, Children Cancer Hospital Egypt (CCHE), Cairo, Egypt; ^6^ Department of Radiation Oncology, National Cancer Institute, Cairo University, Children Cancer Hospital Egypt (CCHE), Cairo, Egypt; ^7^ Department of Radiodiagnosis Oncology, National Cancer Institute, Cairo University, Children Cancer Hospital Egypt (CCHE), Cairo, Egypt; ^8^ School of Global Public Health, The American University in Cairo (AUC), Cairo, Egypt; ^9^ Department of Surgical Oncology, National Cancer Institute, Cairo University, Children Cancer Hospital Egypt (CCHE), Cairo, Egypt

**Keywords:** bilateral, Wilms’ tumor, synchronous, nephron-sparing surgery, children, Egypt, CCHE, 57357

## Abstract

**Introduction:**

Bilateral Wilms’ tumor (BWT) represents 5 – 8% of patients with Wilms’ tumor (WT). Despite the improvement of WT survival, still patients with BWT are challenging with a high risk of relapse and renal failure.

**Purpose:**

To assess the outcome of pediatric patients with BWT treated at Children’s Cancer Hospital Egypt (CCHE) 57357, and to assess the long-term nephrotoxicity among these patients.

**Methods:**

This was a retrospective study including all patients with synchronous BWT treated at CCHE 57357 from 2007 until 2020. Records of all patients were reviewed. Patients were followed up until end of December 2024.

**Results:**

In 115 eligible patients, median age was 31.5 months (range 3 month – 9 years). Male to female ratio was 1:1.67. Abdominal mass was the most common presentation (69.5%). At the end of the study, 5-year EFS and OS were 69% and 76.7%. Patients with high-risk pathology had the worst prognosis regarding EFS and OS, keeping its significance in uni- and multivariate analyses as the factor with highest hazard ratio (HR = 2.931, 95% CI 1.366 - 6.289 p 0.006). Out of 29 events related to the disease, 21 included local progression/relapse (72.5%), and 8 lung progression/relapse (27.5%). The incidence of ESRD was 6.9% (8 patients).

**Conclusion:**

Bilateral Wilms’ tumor poses a significant challenge all over the world. Proper timing and type of surgery should be tailored according to each patient. Low-middle income countries face additional challenges related to supportive care for those children who usually present at a very young age with a tangible proportion of them requiring regular hemo dialysis and occasionally renal transplantation.

## Introduction

Wilms’ tumor (WT) represents 90% of childhood renal cancers and bilateral Wilms’ tumor (BWT) represents 5-10% of all patients with WT. Patients with BWT usually present with synchronous tumors, but around 1% of patients with unilateral WT will develop metachronus WT in the contralateral kidney (1). BWT is more common in females and presents at an earlier age than unilateral WT (2).

Both Children’s Oncology Group (COG) and Societe Internationale D’oncologie Pediatrique (SIOP) recommend 6–12 weeks of preoperative chemotherapy to shrink tumors and enable nephron-sparing surgery (NSS). The COG AREN0534 trial demonstrated that 84% of patients underwent surgery within 12 weeks (3). SIOP protocols similarly emphasize completing surgery within 12 weeks, as prolonged chemotherapy (>120 days) correlates with increased relapse risk in non-metastatic BWT (4).

A comparative analysis of treatment centers across income levels revealed that bilateral NSS was performed more frequently in high-income settings. This discrepancy may reflect advanced disease presentation in low-income centers, where tumors were larger (median stage III vs. stage II) at diagnosis. The findings suggest that observed mortality disparities likely arise from patient-specific factors (e.g., late presentation with median age at diagnosis 34 months for low-income centers and 24 months for high-income centers) rather than gaps in medical infrastructure between regions. Existing large-scale studies on BWT lack representation from diverse healthcare settings across developed and developing regions. This limitation prevents robust international comparisons of treatment protocols and outcomes in the current evidence base (1). In the current work, we demonstrate the clinical outcome and long-term nephrotoxicity of the largest cohort of patients with BWT from a single center.

## Materials and methods

### Study population

This was a retrospective study, including all pediatric patients with synchronous BWT treated at Children’s Cancer Hospital Egypt (CCHE 57357) from July 2007 until the end of December 2020. Patients were followed until the end of December 2024. Patients with unilateral Wilms’ tumor and contralateral nephroblastomatosis and patients with metachronous BWT were excluded from this study.

Records of all patients were reviewed for their demographic and clinical characteristics, investigations done (Imaging, pathology reports), type and time of surgery, treatment received (chemotherapy +/- radiotherapy), response to treatment, and acute and chronic nephrotoxicity. Tumor staging was revised according to COG clinico-pathologic staging system of Wilms’ tumor (5).

### Clinical treatment protocol

For all patients, initial diagnostic work-up included a CT scan with contrast of the chest, abdomen and pelvis to assess the nature, extent and relations of bilateral renal masses and check for the presence of other metastatic lesions (e.g. nodal, hepatic or pulmonary). Histologic confirmation was not required for diagnosis, nonetheless, patients who underwent Tru-cut biopsy initially for pathology confirmation were included in the study. All patients were treated according to the CCHE-BWT protocol adopted from the AREN0534 COG study (3). Following radiological confirmation of the diagnosis, patients received 6 weeks of neoadjuvant chemotherapy consisting of a three-drug regimen (vincristine, actinomycin-D, and doxorubicin (VAD)).

Response assessment by CT was done after week 6 according to revised RECIST criteria version 1.1 (6). Target lesions were defined as lesions greater than 10 mm. The response was defined according to the least responsive kidney as complete remission (CR) if there was no evidence of disease clinically and on imaging, partial regression (PR) if there was at least a 30% decrease in the sum of diameters of target lesions, progressive disease (PD) if there was ≥20% increase in the sum of diameters in one or more lesions or appearance of new lesions, stable disease (SD) if there was neither sufficient shrinkage to qualify for PR nor sufficient increase to qualify for PD. Relapse was defined as an evidence of disease recurrence after achieving complete remission.

After 6 weeks of VAD chemotherapy, our dedicated surgical team, with sub-specialized surgeons in renal tumors and nephron-sparing surgery (NSS), evaluated whether NSS, such as a partial nephrectomy or tumor enucleation, was a viable option for at least one kidney. All surgical procedures were performed at our center by our surgical oncology team. If PR was achieved, but NSS was still not feasible, neoadjuvant VAD was continued for six more weeks. After 12 weeks of neoadjuvant chemotherapy, surgical exploration was attempted for all cases, and if NSS was still not feasible, bilateral open biopsies were obtained for histopathologic assessment (**Supplementary Table 1**).

Adjuvant chemotherapy and radiotherapy were determined according to the higher stage and more aggressive pathology between both kidneys (**Supplementary Table 2**). The tumor pathology was categorized into three risk groups: low risk (LR) for tumors displaying complete necrosis; intermediate risk (IR) for all histological subtypes except those with complete necrosis, anaplasia, or blastemal predominance; and high risk (HR) for tumors exhibiting anaplasia or blastemal predominance observed after chemotherapy. Blastemal predominance was defined as presence of < 66% tumor necrosis, with blastemal component comprising more than two third of viable tumor (7). Anaplasia was defined by the presence of atypical tri- and multipolar mitotic figures, marked nuclear enlargement, and hyperchromatism. Diffuse anaplasia was considered in multi-focal anaplasia, anaplasia in an extrarenal site, in a random biopsy specimen, or the presence of focal anaplasia and marked nuclear unrest elsewhere in the tumor (7). In our cohort, all patients with focal anaplasia (n =3) presented with an advanced disease, necessitating aggressive chemotherapy. Two patients presented with stage IV disease and one patient showed blastemal predominance in the contralateral kidney.

Event free survival (EFS) is defined as the time from date of diagnosis to date of relapse, progression, death or last follow up. Overall survival (OS) is defined as the time from date of diagnosis to date of death or last follow up. Acute kidney injury (AKI) was defined according to the Kidney Disease: Improving Global Outcomes (KDIGO) criteria as serum creatinine ≥3.0 times baseline, increase in serum creatinine to ≥4.0 mg/dL, initiation of renal replacement therapy, decrease in eGFR to <35 mL/min per 1.73 m2, <0.3 mL/kg/h for ≥24 h, or anuria for ≥12 h (8), and chronic kidney disease was labelled and graded according to Common Terminology Criteria for Adverse Events (CTCAE) Version 5.0 as grade 1 if eGFR 60 ml/min/1.73 m2, grade 2 if eGFR 59–30 ml/min/1.73 m2, grade 3 if eGFR 29–15 ml/min/1.73 m2, and grade 4 (ESRD): eGFR less than 15 ml/min/1.73 m2 (9). In our study, the eGFR was calculated using the Schwartz equation: eGFR = (0.41 x height in cm)/serum Cr in mg/dL (10). As for the regular follow-up of patients with BWT to detect ESRD, it was routinely done by the primary pediatric oncologist during clinic visits and checking the renal function tests including serum creatinine. Whenever there’s an elevation in the serum creatinine or any other signs of ESRD, the patient is referred to our nephrology clinic. It’s noteworthy that all patients who required renal transplantation were referred outside our center to a renal transplantation center.

### Statistical analysis

Statistical analysis was done using IBM SPSS^®^ Statistics version 22 (IBM^®^ Corp., Armonk, NY, USA). Numerical data was expressed as mean, standard deviation, and median. categorical data was expressed as frequencies and percentages. Survival analysis was done using Kaplan-Meier method and Log rank test was used in comparing survival rates. Hazard Ratios (95% CI) were calculated through univariate and multivariate Cox regression modeling to examine the relationships between the survival and different covariates; syndromic features (yes/no), residual (yes/no), microscopic margin (yes/no), metastatic status (present/absent), local stage (I&II/III), final stage (I&II/III&IV), pathology (LR&IR/HR). A p-value < 0.05 was considered significant throughout the analysis.

## Results

### Patient characteristics

During the study period, 1264 patients with WT were treated at CCHE. A total of 127 patients presented with bilateral renal lesions suggestive of BWT. Twelve patients were excluded from the study; seven patients had unilateral WT with contralateral nephroblastomatosis, four patients were considered as unilateral WT with contralateral renal cyst, and one patient lost follow up after 2 weeks of presentation. Patients with synchronous BWT constituted around 9% of all patients with WT (115/1264).

**Table 1** illustrates the clinical characteristics of the included patients. Out of the 115 patients included, 43 were males with a male to female ratio of 1:1.67 and the mean age was 31.5 months (range 3 months – 9 years). Abdominal swelling/mass was the most common presenting sign and symptom reported in 80 patients (69.5%), followed by hypertension 17 (14.7%) which were discovered during routine assessment of vital signs and clinical examination. Gross hematuria was observed in 5 patients (4%). Congenital anomalies/syndromes were clinically detected in 19 patients (16.5%) (**Table 2**).

**Table 1:** Demographic and clinical characteristics of the studied patients.

Patients' characteristics	Number	Percent
Gender	
Male	43	37.4%
Female	72	62.6%
Stage (2 patients died before surgery without definite staging)	
Stage I	19	16.8%
Stage II	20	17.7%
Stage III	55	48.7%
Stage IV	19	16.8%
Common Disease Presentations	
Abdominal Swelling	80	69.5%
Hypertension	17	14.7%
Abdominal Pain	11	9.5%
Fever	8	7%
Gross Hematuria	5	4%
Vomiting	4	3.4%
Acute kidney injury	2	1.7%

**Table 2:** Clinical characteristics of syndromic patients.

Syndromic patients' characteristics	Number
Gender	
Male	7
Female	12
Mean Age	
32 months	
Diagnoses	
Beckwith-Wiedemann Syndrome	3
WAGR*	2
Denys-Drash Syndrome	2
Fanconi Anemia**	2
Bilateral undescended testicle	3
Isolated hemihypertrophy	1
Horseshoe Kidney	1
Mental Retardation	2
Dysmorphic features	2
Absent eyeballs	1

*Wilms tumor, aniridia, genitourinary anomalies, and a range of developmental delays.

**DNA stress test confirmed the clinical suspicion.

### Tumor response

After 6 weeks of neoadjuvant chemotherapy, 85 patients (75.2%) achieved PR, 21 patients (18.5%) showed SD, 7 patients (6.2%) had PD, while 2 patients died before week 6 due to huge tumor burden and late presentation.

**Figure 1:**
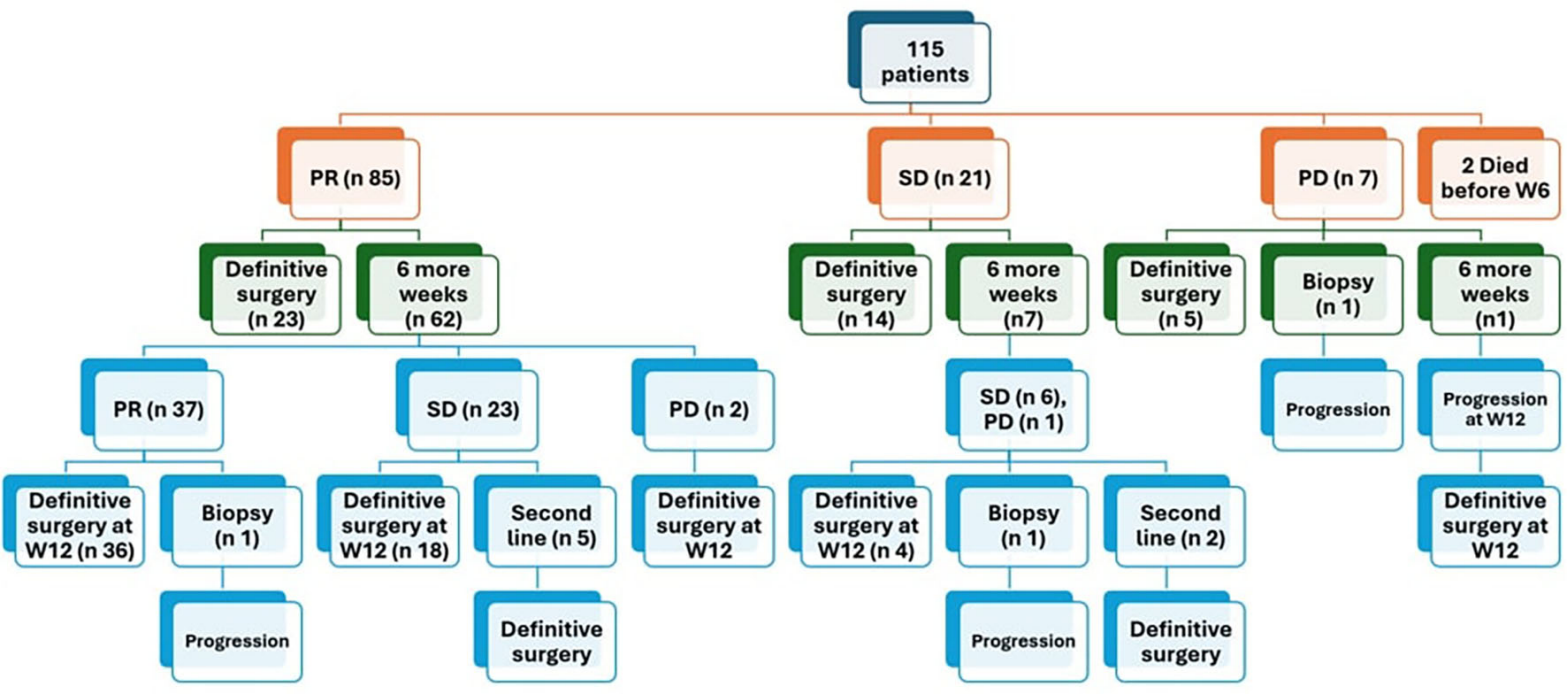
Response of patients after 6 weeks of chemotherapy PR partial response, SD stable disease, PD progressive disease.

**Figure 2:**
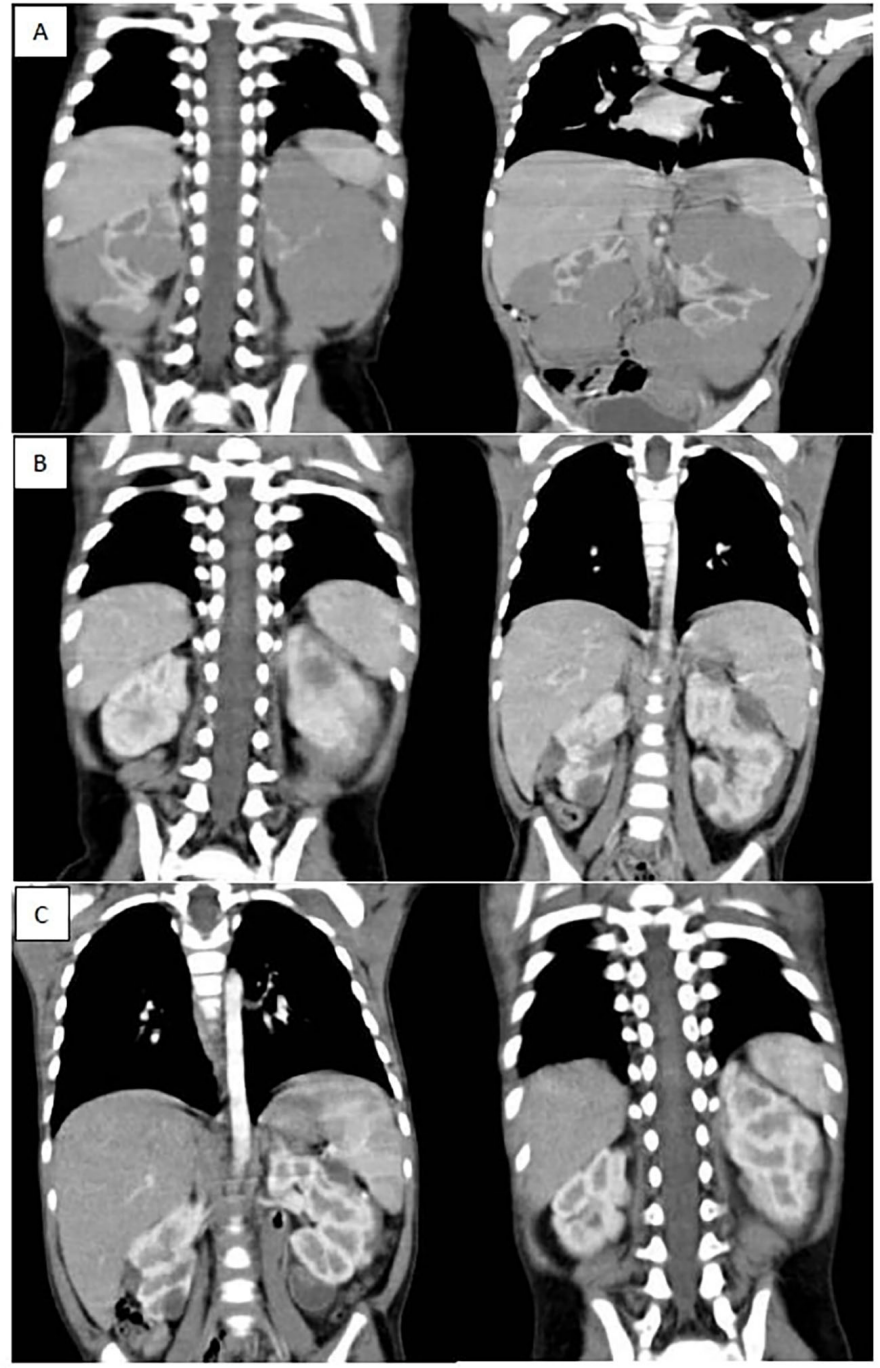
**(A)** shows bilateral renal masses with the left kidney diffusely infiltrated with a huge mass while the right kidney shows multiple variable sized mass lesions, **(B)** shows marked regression of the bilateral renal masses after 6 weeks of neoadjuvant chemotherapy (average 90 % reduction of target lesions), **(C)** shows further regression of bilateral renal masses after 12 weeks of neoadjuvant chemotherapy.

Out of 85 patients who achieved PR, 62 patients (72.9%) received 6 more weeks of chemotherapy and the response after 12 weeks chemotherapy showed that 59.5% of them (37/62) achieved further regression, while none of those who had SD (7/21) or PD (1/7) after 6 weeks showed any regression after addition of 6 more weeks. Of these 28 patients, 8 patients exhibited PR in the contralateral kidney. For those specific cases, our multidisciplinary team opted to administer an additional six weeks of neoadjuvant chemotherapy in the hope of achieving further response in the responding kidney to facilitate NSS for this kidney. However, this approach proved ineffective. (**Figures 1**, **2**).

### Definitive surgery and radiotherapy

**Figure 3:**
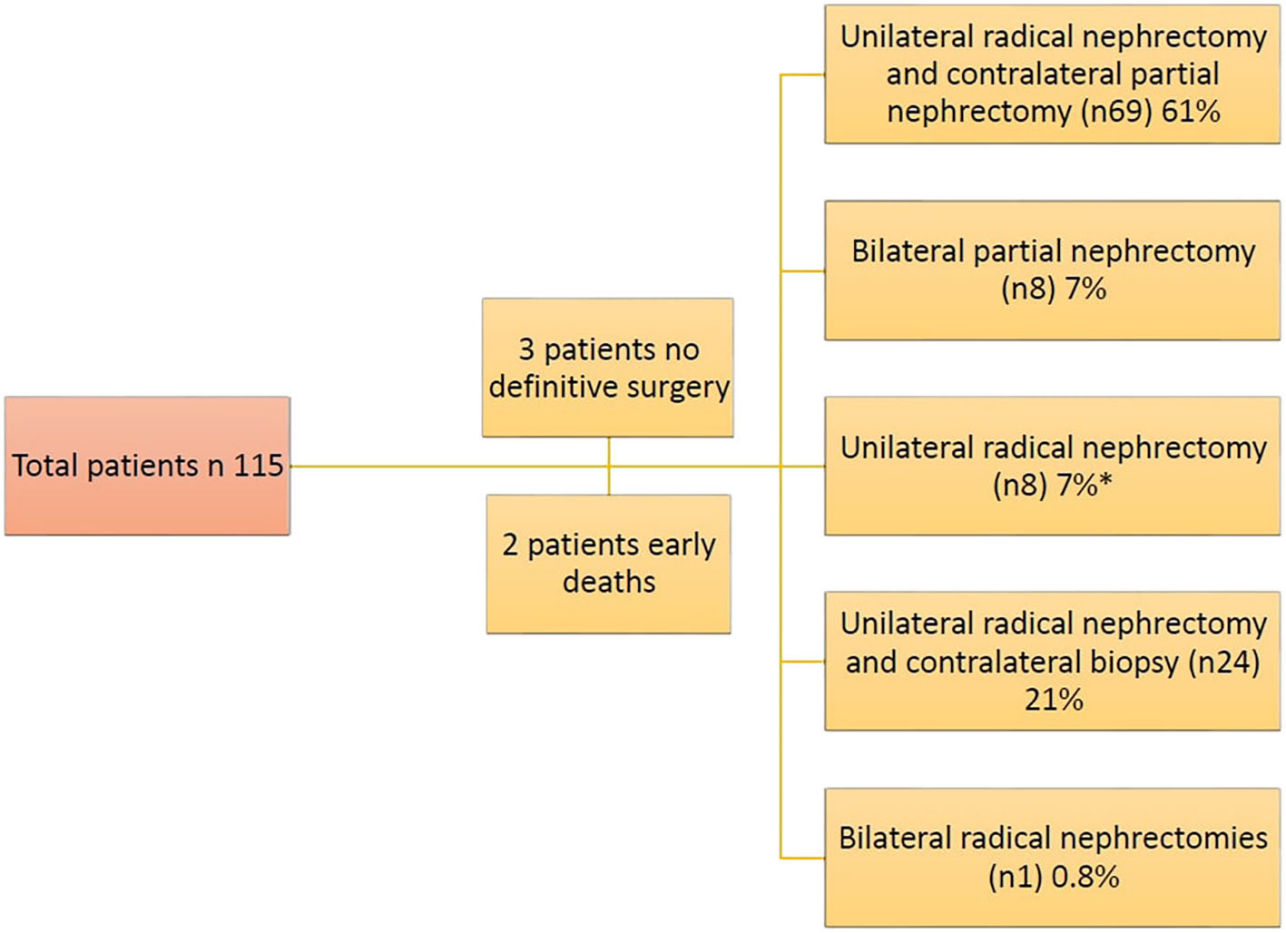
Types of surgical approaches for bilateral Wilms’ tumors in our cohort (* Intraoperative US showed a scar with no residual mass (achieved CR in one kidney)).

**Figure 4:**
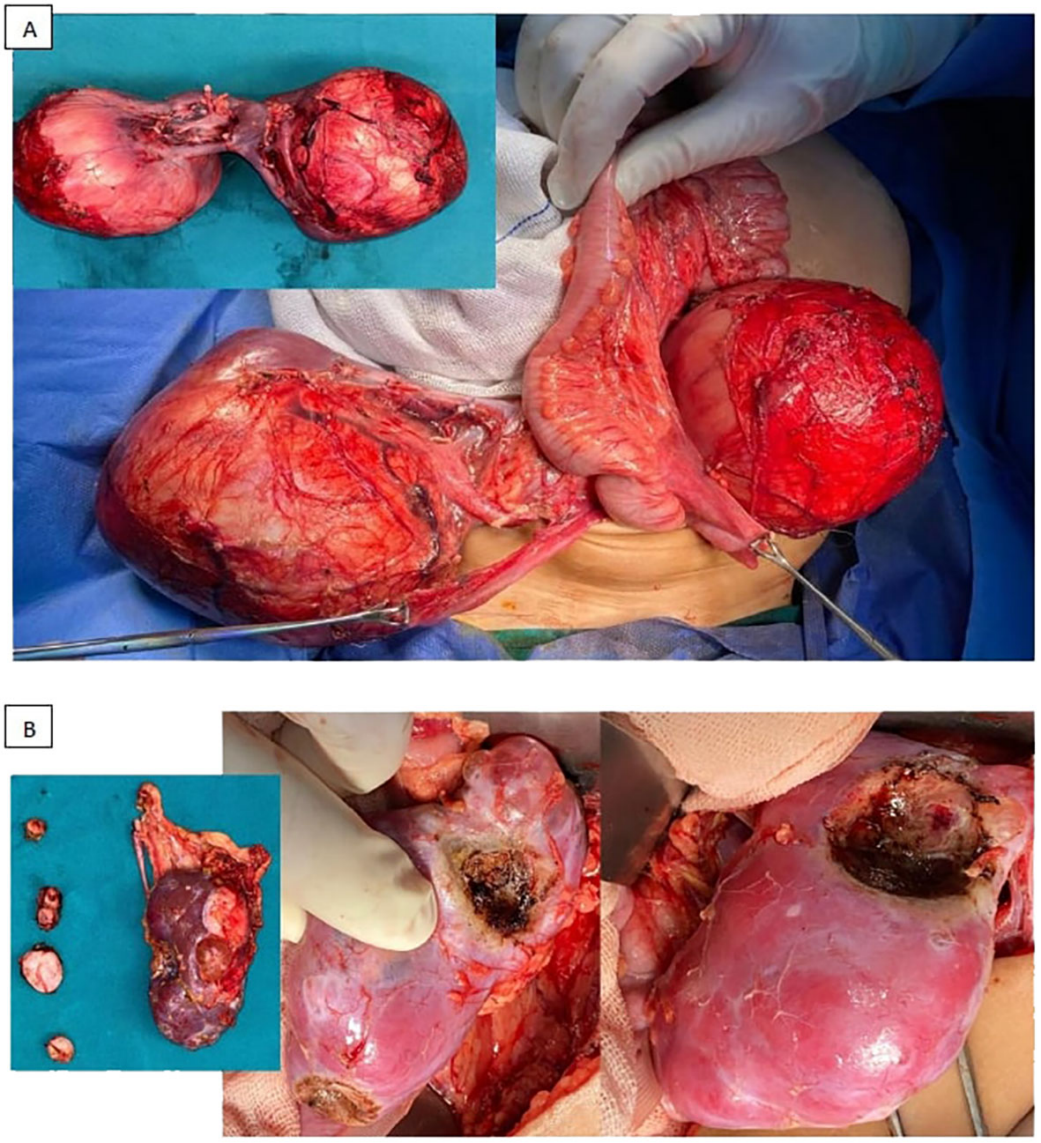
Examples for different surgical approaches [**(A)** bilateral radical nephrectomies, **(B)** radical nephrectomy and NSS (in the form of multiple enucleations)].

Three patients didn’t undergo definitive surgery at all, due to inadequate response post neoadjuvant chemotherapy. They only had open biopsies which showed anaplastic histology in 1 patient and favorable histology in 2 patients, and eventually the disease progressed on chemotherapy, and they succumbed to their disease. Out of 110 patients had surgery, 69 patients (61%) had unilateral radical nephrectomy with contralateral NSS, 8 patients (7%) had bilateral NSS, while 24 patients (21%) had unilateral radical nephrectomy with contralateral biopsy. Eight patients (7%) had unilateral radical nephrectomy, and the intraoperative US showed a scar with no residual mass (achieved CR in one kidney) (**Figures 3**, **4**). Forty patients didn’t undergo LN sampling during the operation, and all were considered as negative based on the results of the initial and postoperative CTs.

A total of 59 patients received local radiotherapy (RT), including 40 patients received flank RT on one side, 13 on both flanks, 6 patients received whole abdominal RT. Twelve patients received concurrent lung radiotherapy for metastatic lung disease, with one of them receiving radiotherapy to the liver for hepatic metastasis. The median time from surgery to radiotherapy was 34 days (range 12–97 days). Patients with favorable histology received 1080 cGy on flank and patients with diffuse anaplasia received 1950 cGy on flank. Lung radiotherapy was delivered with a dose of 1200 cGy and an additional boost dose of 750 cGy if the patient had gross residual at end of treatment.

### Histopathological examination

Analysis of histopathologic subtypes in relation to preoperative radiological response (**Table 3**) revealed that approximately 50% of patients with SD at week 6 evaluation (10 out of 21) and nearly all patients with PD (6 out of 7) exhibited stromal predominance. Postoperative staging analysis (**Table 4**) demonstrated that most patients with LR-IR pathology (73/82, 89%) exhibited locoregional non-metastatic disease. Notably, 50% of patients with anaplastic histology presented metastatic disease (5/10).

**Table 3:** Correlation between response to chemotherapy and type of pathology.

Response at week 6 evaluation	PR	SD	PD	Total
Complete therapy response	2	–	–	2
Mixed type	42	6	–	48
Epithelial	6	–	–	6
Stromal	9	10	6	25
Teratoid	-	1	–	1
Blastemal	19	2	–	21
Focal anaplasia	3	–	–	3
Diffuse anaplasia	4	2	1	7
Total	85	21	7	113

PR, partial regression; SD,stable disease; PD, progressive disease.

**Table 4:** Histopathological subtypes classified by disease stage.

Stage	LR and IR pathology	Blastemal predominance	Focal Anaplasia	Diffuse Anaplasia	Total
Stage I	17	2	0	0	19
Stage II	14	6	0	0	20
Stage III	42	8	1	4	55
Stage IV	9	5	2	3	19
Total	82	21	3	7	113

LR, low risk; IR, intermediate risk.

### Clinical outcomes

The median follow-up period was 67 months (range 3.55 to 181.3 months). At the end of the study, 29 patients died. The 5-year EFS of the whole cohort was 69% and 5-year OS was 76.7% excluding the 2 early deaths (**Tables 5**–**7**).

**Table 5:** Event-free survival (EFS) and overall survival (OS) and their relation to the prognostic factors.

Prognostic factors	No.	No of events	5-year EFS (%)	p- value	No of deaths	5-year OS (%)	p-value
Whole group			69%			76.7%	
Age							
2 years andbelow	57	15	73.2%	0.296	13	76.8%	0.57
Above 2 years	58	21	61.9%		16	73.5%	
Syndromes							
No	96	29	69.5%	0.358	20	79.8%	0.005
Yes	19	7	59.6%		9	52.6%	
Final stage							
Stage I and II	39	8	79.2%	0.115	6	84.1%	0.124
Stage III and IV	74	26	63.6%		21	72.7%	
Metastatic status							
No	96	28	69.9%	0.297	23	76.9%	0.472
Yes	19	8	57.9%		6	67.0%	
Local stage							
Stage I and II	43	8	81.2%	0.04	6	88.1%	0.05
Stage III	70	26	61.3%		21	69.7%	
Pathology*							
LR and IR	81	17	78.2%	0.001	13	84.9%	0.004
HR	31	16	47.0%		13	57.9%	
Gross residual lesion							
No	83	18	77.7%	0.001	15	81.7%	0.009
Yes	30	16	45.4%		12	62.5%	
Margin status							
Negative	53	10	81.0%	0.002	7	86.4%	0.014
Positive	30	8	71.6%		8	73.3%	
Gross residual	30	16	45.4%		12	62.5%	
Upfront Tru cut biopsy							
No	90	26	70.2%	0.360	21	77.8%	0.334
Yes	25	10	59.4%		8	68.0%	

(* one patient had teratoid WT wasn’t included, and the 2 early deaths included in age, syndromic patients, and metastatic status analysis to reach a total of 115 patients).

**Table 6:** Cox regression-EFS and OS of the whole cohort (uni-variate analysis).

Prognostic factors	No.	Cox regression-EFS (uni-variate)				Cox regression-OS (uni-variate)			
		HR	95% CI		p- value	HR	95% CI		p-value
			Lower	Upper			Lower	Upper	
Syndromes									
No	96	1.000	0.643	3.360	0.361	1.000	1.342	6.503	0.007
Yes	19	1.470				2.954			
Local stage									
Stage I/II	43	1.000	1.006	4.915	0.048	1.000	0.972	5.984	0.058
Stage III	70	2.224				2.412			
Metastatic status									
No	96	1.000	0.690	3.327	0.300	1.000	0.565	3.418	0.474
Yes	19	1.515				1.390			
Final stage (2 groups)									
Stage I/II	39	1.000	0.847	4.134	0.121	1.000	0.810	4.986	0.132
Stage III/IV	74	1.871				2.010			
Pathology									
LR and IR	81	1.000	1.521	6.010	0.002	1.000	1.356	6.326	0.006
HR	31	3.023				2.929			
Gross residual lesion									
No	83	1.000	1.584	6.106	0.001	1.000	1.235	5.697	0.012
Yes	30	3.110				2.652			

HR Hazard ratio, CI confidence interval.

**Table 7:** Cox regression-EFS and OS of the whole cohort (multi-variate analysis).

Prognostic factors	No.	Cox regression-EFS (multi-variate)				Cox regression-OS (multi-variate)			
		HR	95% CI		p- value	HR	95% CI		p-value
			Lower	Upper			Lower	Upper	
Syndromes									
No	94	1.000	0.433	2.758	0.851	1.000	1.055	6.074	0.037
Yes	18	1.093				2.532			
Local stage									
Stage I/II	43	1.000	0.353	21.820	0.331	1.000	0.230	21.272	0.493
Stage III	69	2.777				2.210			
Metastatic status									
No	93	1.000	0.458	3.246	0.692	1.000	0.491	4.654	0.471
Yes	19	1.219				1.512			
Final Stage (2 groups)									
Stage I/II	39	1.000	0.046	3.506	0.411	1.000	0.035	4.271	0.437
Stage III/IV	73	0.404				0.385			
Pathology									
LR and IR	81	1.000	1.366	6.289	0.006	1.000	1.356	5.733	0.032
HR	31	2.931				2.929			
Gross residual lesion									
No	83	1.000	1.242	6.676	0.014	1.000	0.809	5.187	0.130
Yes	29	2.879				2.490			

HR Hazard ratio, CI confidence interval.

**Figure 5:**
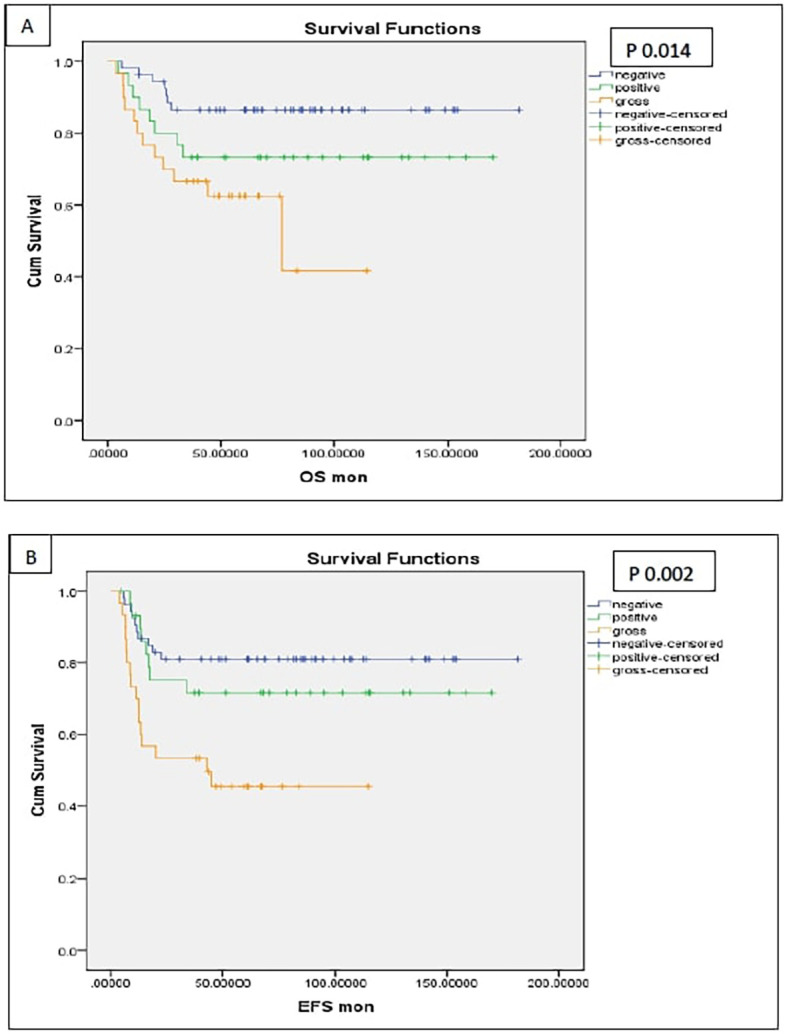
Survival of patients with different margin status [**(A)** OS, **(B)** EFS].

Patients with postoperative gross residual disease demonstrated significantly reduced 5-year EFS and OS compared to those without residual disease (EFS: 45.4% vs. 77.7%, p 0.001; OS: 62.5% vs. 81.7%, p 0.009) (**Figure 5**). Univariate and multivariate analyses identified gross residual disease as an independent predictor of poorer EFS (Hazard ratio = 2.879, 95% CI: 1.242–6.676, p 0.014), though this association did not retain statistical significance for OS (Hazard ratio = 2.490, 95% CI: 0.809–5.187, p 0.13).

**Figure 6:**
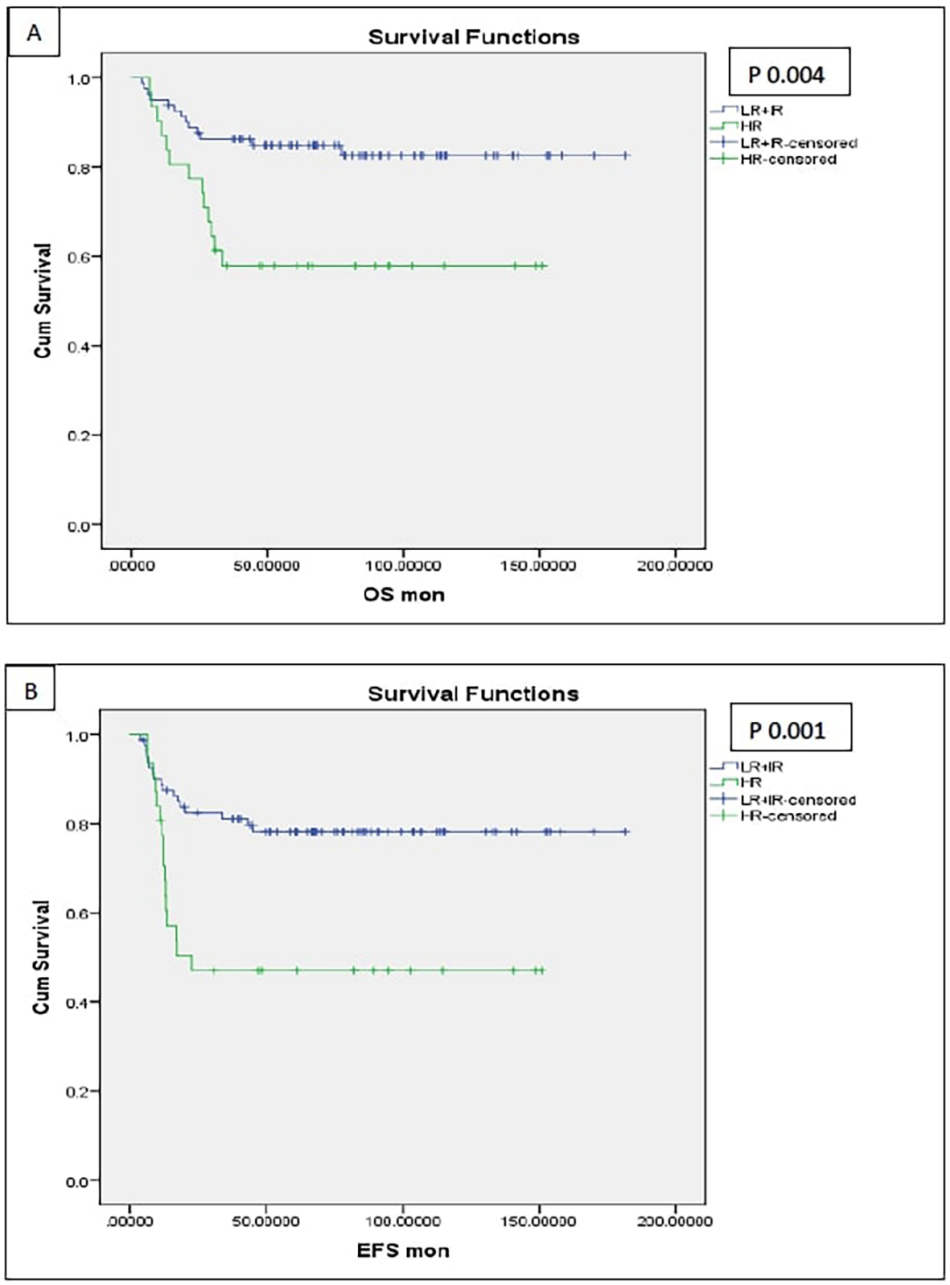
Survival of the patients with high-risk pathology (anaplasia and blastemal) [**(A)** OS, **(B)** EFS].

**Figure 7:**
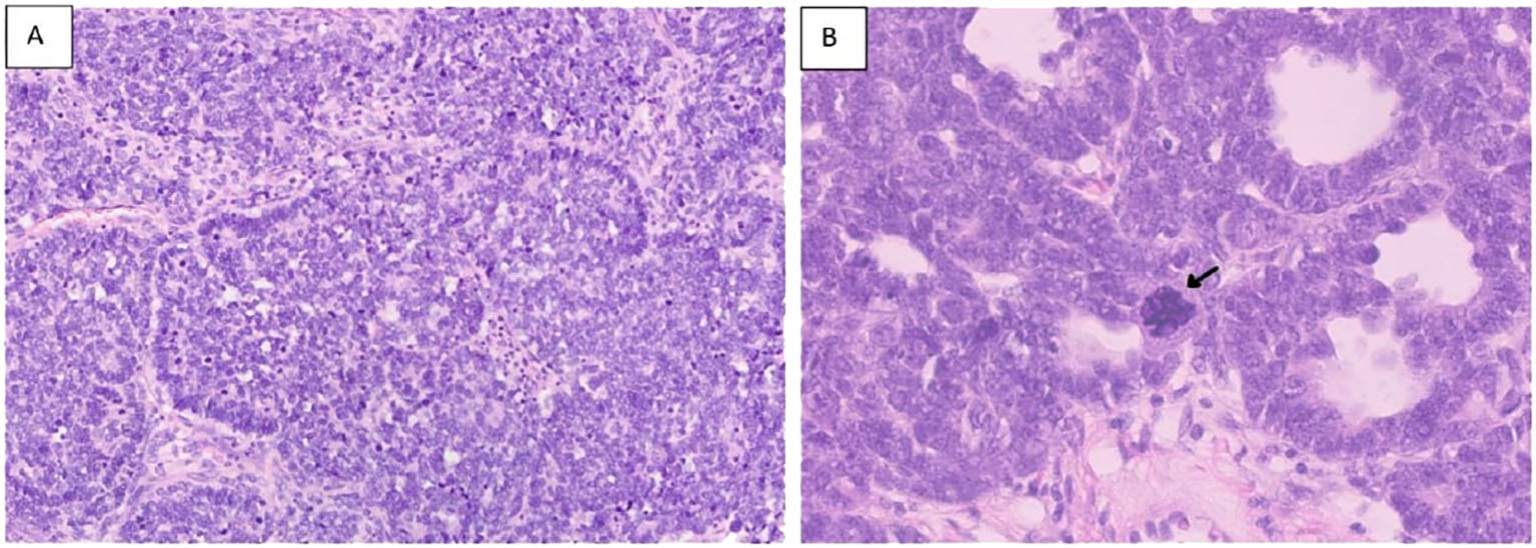
Hematoxylin and eosin stains [**(A)** section showing predominance of primitive cells with hyperchromatic nuclei and scanty cytoplasm (Blastemal predominance) X20.] [**(B)** showing large anaplastic cell with large hyperchromatic multipolar mitotic figure (arrow) 40X].

Patients with HR pathology exhibited significantly poorer 5-year outcomes compared to LR and IR groups, with EFS rates of 47% vs. 78.2% (p 0.001) and OS rates of 57.9% vs. 84.9% (p 0.004) (**Figures 6**, **7**). In both univariate and multivariate analyses, high-risk pathology remained a significant independent prognostic factor, showing the highest hazard ratio of 2.931 (95% CI: 1.366–6.289; p 0.006).

Analysis of patients stratified by local disease stage indicated that those with stage III tumors had poorer EFS compared to stages I and II (61.3% vs. 81.2% p 0.04). However, this association between local stage and survival outcomes lost statistical significance in multivariate analysis (p 0.331).

Syndromic patients demonstrated markedly reduced 5-year OS compared to non-syndromic counterparts (52.6% vs. 79.8%, p 0.005). In multivariate analysis, syndromic status emerged as an independent prognostic factor alongside high-risk pathology, with a hazard ratio of 2.532 (95% CI: 1.055–6.074, p 0.037).

The median interval from initial presentation to disease progression or relapse was 15.1 months (range: 5.7–45.1 months). Out of 36 events, 29 were progression/relapse events with 51.7% (15 patients) developing local failure, 20.6% (6 patients) developing local and distant disease, while 27.5% (8 patients) developed pulmonary recurrence. Local events manifested later (median: 16.5 months) compared to lung-related events (median: 11.7 months).

Among the cohort, 29 deaths occurred, with 19 deaths disease related, and 10 non-disease related. Among these 10 fatalities, six patients had clinical syndromes, two experienced early deaths (occurred during the first chemotherapy cycle in infants aged 11 and 12 months at presentation), one succumbed to postoperative complications, and one died from COVID-19 infection.

### Long term follow up of kidney functions

**Figure 8:**
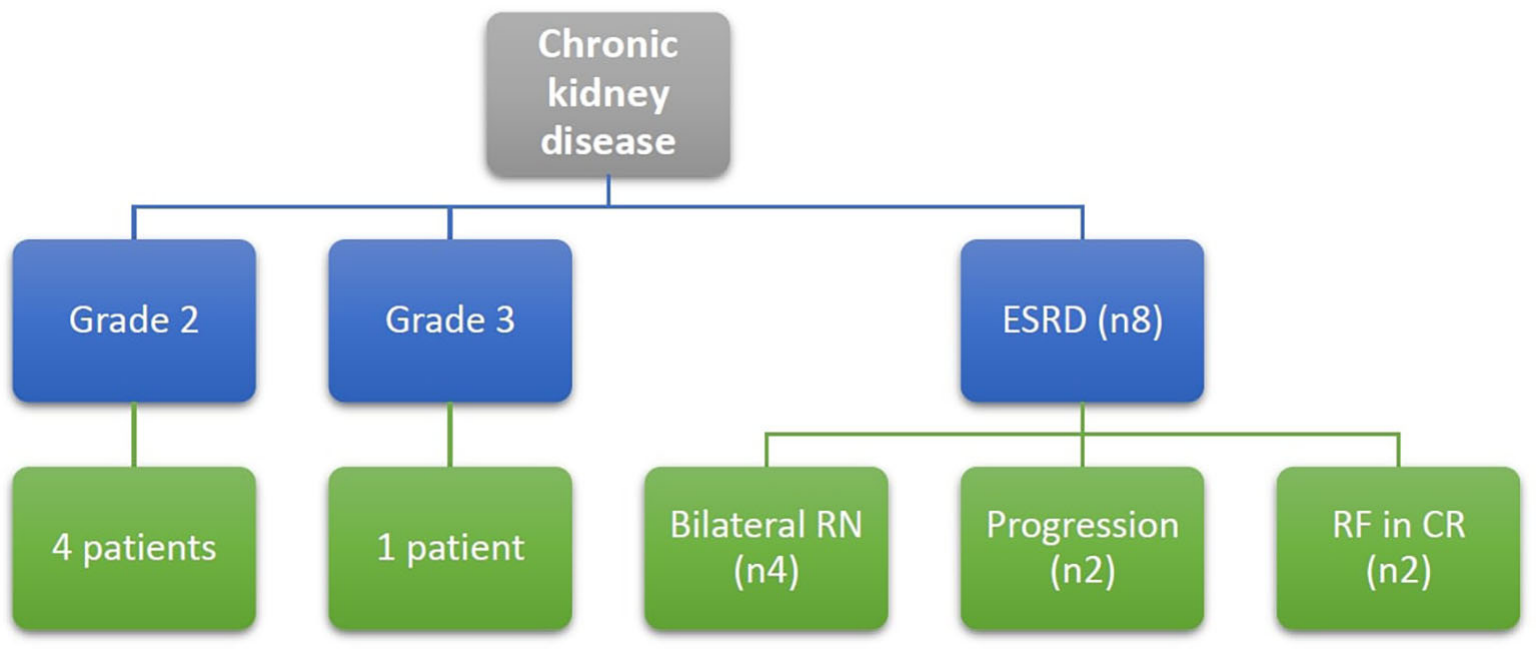
Patients with chronic kidney disease at end of the study defined according to CTCAE version 5, Grade 2: eGFR 59-30 ml/min/1.73m2, Grade 3: eGFR 29-15 ml/min/1.73m2, ESRD: eGFR below 15 ml/min/1.73m2, RF, renal failure; RN, radical nephrectomy.

As per CTCAE version 5, four patients were diagnosed with chronic kidney disease (CKD) grade 2, and one patient had CKD grade 3. Eight patients (6.9%) developed ESRD, necessitating regular dialysis (**Figure 8**).

Among these 8 patients with ESRD, 3 patients had bilateral radical nephrectomy due to disease progression requiring subsequent removal of the remaining kidney, and one patient underwent bilateral nephrectomy as a single-stage procedure. Of those four patients, one patient received a successful renal transplant, while another child was still on dialysis at the end of study date since parent refused renal transplantation. Two fatalities occurred while on dialysis, one of them was due to COVID-19 infection.

Among the remaining 4 patients, 2 patients developed renal failure several years after initial treatment (3- and 8-years post-follow-up), both underwent successful renal transplantation. Finally, two patients had disease relapse and progressed to ESRD despite second-line chemotherapy, and both required dialysis as part of palliative care for uncontrolled disease.

## Discussion

This study represents the largest single-institution investigation of BWT to date, involving 115 patients diagnosed with synchronous BWT who received care at CCHE 57357 (**Supplementary Table 3** includes different studies reporting the outcome of patients with BWT until the current study date 11–22). The mean age of patients in our cohort was 31.5 months (range: 3 months to 9 years). A systematic review and meta-analysis reported a comparable mean diagnostic age of 28 ± 5.92 months (23). Congenital anomalies or syndromes were clinically identified in 19 patients (16.5%) in the current cohort. This proportion is lower than the 22% rate (120 out of 545 patients) observed in a 2017 review study (2).

Assessment of treatment response following 6 weeks of neoadjuvant chemotherapy revealed that majority of patients (75%) achieved PR in the least responsive kidney, which is consistent with outcomes reported in the AREN0534 COG trial where almost 70% of patients attained CR and PR. Conversely, PD incidence was higher in our cohort (6.2%, 7/113) compared to the COG trial where only 2 patients (~1%) had disease progression.

Among 85 patients with PR at the week 6 evaluation, 62 patients (72.9%) received an additional 6 weeks of chemotherapy, with 59.5% of these patients (37/62) achieved further tumor regression. In contrast, none of the patients with SD (7/21) or PD (1/7) at the week 6 evaluation exhibited regression after addition of 6 more weeks of neoadjuvant chemotherapy underscoring the importance of local control rather than further chemotherapy for those who didn’t achieve a good response after 6 weeks of neoadjuvant chemotherapy.

Correlation between radiological response to neoadjuvant chemotherapy and histopathologic subtypes revealed that approximately 50% of patients with SD (10 out of 21) and nearly all patients with PD (6 out of 7) at week 6 evaluation, exhibited stromal predominance. These findings align with established patterns of poor chemoresponsiveness in stromal-dominant tumors, as evidenced by the COG AREN0534 trial, where 18 of 21 patients with stromal pathology failed to achieve PR post-chemotherapy. Notably, survival rates remained consistent regardless of treatment responses or disease stages within each histologic category (24).

In our study, after 12 weeks of chemotherapy, definitive surgery was done for 103 patients (91.1%). This is comparable to the percentage reported in COG clinical trial (84%).

Our study demonstrated a higher rate of unilateral radical nephrectomy with contralateral partial nephrectomy 61% (69/113) and lower rate of bilateral partial nephrectomy 7% (8/113) compared to what was reported in the COG trial 48% and 35% respectively. This discrepancy reflects delayed diagnosis and locally advanced disease at presentation, which restricted the feasibility of bilateral NSS as a definitive treatment option.

In our cohort, the 5-year EFS and OS rates were 69% and 76.7%, respectively. These outcomes aligned with those reported in the Associazione Italiana Ematologia Oncologia Pediatrica (AIEOP) study, which evaluated 93 patients with BWT and documented 4-year DFS and OS rates of 66.5% and 80%, respectively. However, our EFS and OS rates were lower than those reported in the COG and SIOP trials. The COG AREN0534 trial reported superior 4-year EFS and OS rates of 82.1% (95% CI: 73.5–90.8%) and 94.9% (95% CI: 90.1–99.7%), respectively. Similarly, the SIOP WT 2001 study demonstrated 5-year EFS and OS rates of 76.1% and 88.1%, with a 10-year OS rate of 84.6% (25, 26).

In both univariate and multivariate analyses, high-risk pathology consistently emerged as an independent and statistically significant predictor of prognosis. These results are aligned with findings from the AIEOP study, which reported 4-year DFS and OS rates of 51% and 62%, respectively, in 18 children with diffuse anaplasia or post-chemotherapy blastemal-type tumors. In contrast, the 67 patients with favorable histology demonstrated superior outcomes, with 4-year DFS and OS rates of 72% and 88% (25).

The association between the local stage and survival outcomes lost statistical significance in multivariate analysis in our study. These findings parallel results from the SIOP WT 2001 Study, where univariate Cox regression analysis of EFS in patients with BWT yielded non-significant hazard ratios for stage II (HR = 1.745, 95% CI: 0.741–4.11), stage III (HR = 1.085, 95% CI: 0.461–2.555), and stage IV (HR = 2.230, 95% CI: 0.923–5.386), with no stage demonstrating statistical significance (p 0.21) (26).

In multivariate analysis, syndromic status was identified as a significant independent predictor of OS, alongside high-risk pathology. This underscores the critical need for enhancing supportive care in syndromic patients. A report from SIOP WT 2001 Study showed that 6 patients with Denys Drash syndrome had ESRD, and bilateral nephrectomy was performed for them.

Eight patients (6.9%) in our study developed ESRD. A study by Breslow et al. found that individuals with BWT face a long-term risk of ESRD nearing 15% over a 15-year follow-up period. Notably, the likelihood of developing ESRD rose substantially in patients who experienced a loss of more than half of their functional kidney tissue (26).

Recent research investigating relapse patterns in patients with BWT reported that most of the relapses occurred after 2 years were to the kidney, proposing that these late-onset occurrences may represent secondary primary malignancies (27). This hypothesis parallels our institutional findings, where localized recurrences manifested a median of five months later than metastatic events.

## Conclusions

BWT remains a global therapeutic challenge, requiring patient-specific surgical timing and approach. Low- and middle-income countries face compounded challenges in managing such patients, usually presenting at very young ages, with locally advanced disease, which necessitates investing in dialysis and renal transplantation infrastructure to address the patients requiring such interventions for optimal survival outcomes.

Continuing neoadjuvant chemotherapy beyond six weeks for patients who did not demonstrate initial good response, did not enhance the likelihood of successful surgical removal of the tumor in these patients. High-risk histopathology exerted the strongest negative impact on both EFS and OS. While gross postoperative residual disease adversely affected EFS, it showed no significant association with OS in multivariate models. Syndromic patients exhibited reduced OS despite comparable EFS rates, underscoring the necessity for enhancing supportive care rather than intensifying therapeutic regimens.

Local treatment failures accounted for over 70% of disease recurrences. Treatment centers must strategically optimize the balance between achieving target EFS rates and managing the projected incidence of ESRD in this patient population.

## Data Availability

The raw data supporting the conclusions of this article will be made available by the authors, without undue reservation.

## Supplementary material

Supplementary Table 1 Preoperative chemotherapy VAD protocol for patients with BWT (COG approach).

Supplementary Table 2 Determination of adjuvant therapy after surgical resection of bilateral Wilms tumor (COG approach).

Supplementary Table 3 Summary of different studies discussing the outcome of patients with BWT.
