# Supplementary material for: Clinical outcome of 115 patients with synchronous bilateral Wilms’ tumor: largest cohort of single-center experience
Source: Front Oncol. 2025 Oct 29;15:1630923. doi: 10.3389/fonc.2025.1630923 (PMC12604981; doi:10.3389/fonc.2025.1630923)
Supplement: Supplementary Table 1 — Preoperative chemotherapy VAD protocol for patients with BWT (COG approach). [file Table1.docx]

Supplementary table (1) Preoperative chemotherapy VAD protocol for patients with BWT (COG approach)


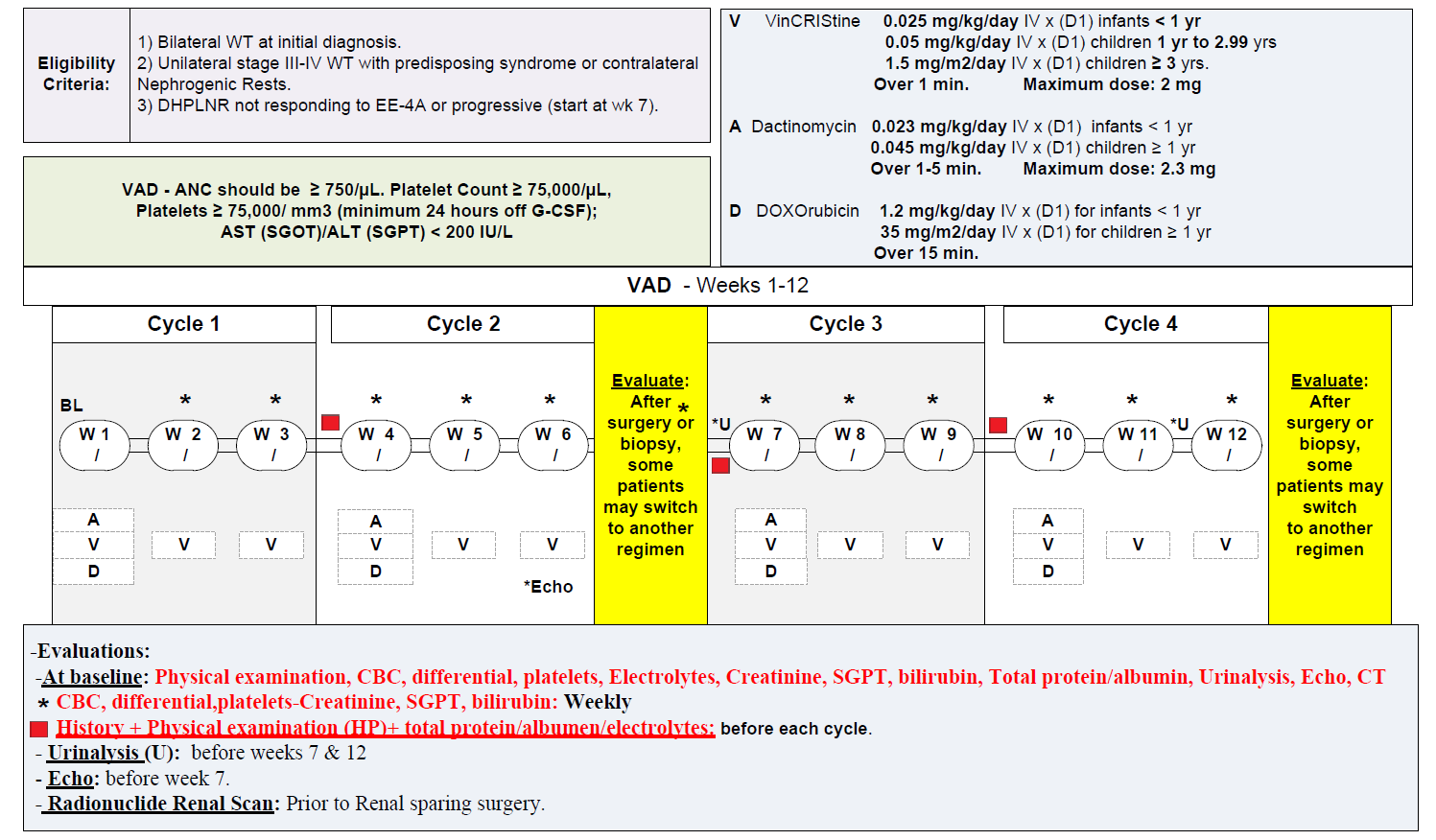


Supplementary table (2) Determination of adjuvant therapy after surgical resection of bilateral Wilms tumor (COG approach)

| Histology | Stage | Adjuvant Regimen |
| --- | --- | --- |
| Completely necrotic | I - II | EE-4A |
|  | III - IV | DD-4A + XRT |
| Intermediate risk | I | EE-4A |
|  | II | DD4A |
|  | III - IV | DD-4A + XRT |
| Blastemal predominant | I | DD4A |
|  | II | Regimen I |
|  | III - IV | Regimen I + XRT |
| Focal anaplasia | I - III | DD-4A + XRT |
|  | IV | Revised UH-1 + XRT |
| Diffuse anaplasia | I | DD-4A + XRT |
|  | II - IV | Revised UH-1 + XRT |

XRT: radiotherapy, EE4A: Vincristine and dactinomycin 19 weeks, DD-4A: Vincristine, dactinomycin, doxorubicin 25 weeks, Regimen I: Vincristine, dactinomycin doxorubicin cyclophosphamide and etoposide 28 weeks, Revised UH-1: Vincristine, dactinomycin doxorubicin cyclophosphamide carboplatin, etoposide 31 weeks (7)

Supplementary table (3): Summary of different studies discussing the outcome of patients with BWT

| N | Country | Year | N. | EFS | OS | ESRD | Ref. |
| --- | --- | --- | --- | --- | --- | --- | --- |
| 1 | UK | 1980-1995 | 70 | - | 69% | 7% | (17) |
| 2 | Turkey | 1980-2013 | 20 | - | 65% | 10% | (18) |
| 3 | South Africa | 1981-2003 | 19 | 29.2%***** | 51.6%***** | 11% | (19) |
| 4 | NWTS-4 (US) | 1986-1994 | 188 | 70% | 84% | 12% | (20) |
| 5 | Brazil | 1987-2022 | 33 | - | 76% | 6% | (21) |
| 6 | SIOP-9/GPOH | 1989-1994 | 28 | 80% | 85% | - | (22) |
| 7 | AIEOP (Italy) | 1990-2011 | 93 | 66.5% | 80% | 1% | (14) |
| 8 | France | 1993-2001 | 49 | 83.4% | 89.5% | 14% | (23) |
| 9 | Egypt | 1993-2008 | 22 |  | 63.5% | 5% | (24) |
| 10 | Japan | 1996-2011 | 31 | 85% | 93% | 13% | (25) |
| 11 | Canada | 2001-2018 | 57 | 80% | 86% | - | (26) |
| 12 | South Africa | 2002-2012 | 20 | - | 85% | 10% | (27) |
| 13 | China | 2008-2022 | 70 | 67.9% | 89.3% | 4.3% | (28) |
| 14 | AREN0534 | 2009-2015 | 189 | 82.1% | 94.16% | - | (7) |
| 15 | SIOP WT 2001 | 2001-2017 | 174 | 76.1% | 88.1% | ** | (13) |
|  | Our study | 2007-2020 | 115 | 69% | 76.7 % | 6.9% |  |

A literature search of PubMed, and google scholar was performed to identify all studies, published after 1990. Keywords (Bilateral, Wilms' tumor, outcome, survival, children) were used. (* for synchronous BWT) (**87% of patients retaining normal renal function without treatment or transplantation)
